# Accuracy of anterior cranial base surfaces acquired from computed tomography imaging

**DOI:** 10.1038/s41598-025-09104-w

**Published:** 2025-07-07

**Authors:** Maurus Kurt Jaeggi, Mohammed Ghamri, Konstantinos Dritsas, Simos Psomiadis, Carlalberta Verna, Demetrios Halazonetis, Nikolaos Gkantidis

**Affiliations:** 1 Department of Restorative, Preventive and Pediatric Dentistry, School of Dental Medicine, University of Bern, Freiburgstrasse 7, 3010 Bern, Switzerland; 2 Department of Orthodontics and Dentofacial Orthopedics, School of Dental Medicine, University of Bern, 3010 Bern, Switzerland; 3 Jeddah Second Health Cluster, 23816 Jeddah, Saudi Arabia; 4 Department of Oral and Maxillofacial Surgery, School of Dentistry, National and Kapodistrian University of Athens, 11527 Athens, Greece; 5 Department of Pediatric Oral Health and Orthodontics, UZB-University Center for Dental Medicine, University of Basel, 4058 Basel, Switzerland; 6 Department of Orthodontics, School of Dentistry, National and Kapodistrian University of Athens, 11527 Athens, Greece

**Keywords:** Skull base, Cone beam computed tomography (CBCT), Trueness and reproducibility, Radiographic imaging accuracy, Three-dimensional imaging, Anatomy, Skeleton, Medical research

## Abstract

Understanding computed tomography scan accuracy is crucial for reliable diagnoses and minimal radiation exposure. This study assessed the accuracy of 3D surface models of the anterior cranial base created using different imaging devices and settings. Ten human skulls were scanned with two cone beam computed tomography (CBCT) scanners, including a low-radiation protocol, a CT scanner, and an optical scanner that provided highly accurate reference models. Water-filled head shells were used to simulate soft tissue during imaging. Reproducibility of the 3D models was evaluated through repeated segmentations, while accuracy was assessed by comparing the segmented models to the reference scans. The primary metric used was the mean absolute distance (MAD) between the 3D surface-approximated models. Results demonstrated high consistency across repeated segmentations, with only minor differences observed (< 0.09 mm). The low-radiation CBCT scans produced 3D models with accuracy comparable to conventional CT scans (median: 0.12 mm, IQR: 0.07 mm). No considerable differences were found between the imaging devices or protocols. These findings confirm that low-radiation CBCT protocols can reliably produce 3D anterior cranial base models comparable to standard CT scans, offering a safer alternative. This supports lower-dose imaging for craniofacial morphology assessment in clinical and research settings, ensuring accuracy while prioritizing patient safety.

## Introduction

The anterior cranial base (ACB) is a key structure of the human skull from several perspectives, such as development and evolution^1,2^, but it is also a major superimposition reference area for the assessment of craniofacial change over time^3–5^. Serial superimposition on the ACB using lateral cephalometric radiographs caries difficulties arising from inherent limitations of two-dimensional (2D) images, such as magnification, distortion and overlapping of neighbouring structures^6^. Therefore, three-dimensional (3D) imaging, mainly computed tomography (CT) and cone beam computed tomography (CBCT), might be preferable^4,7^. Clinicians and researchers are increasingly interested in investigating 3D imaging modalities and superimposition techniques, to ensure realistic virtual patient models, or better assess pathology, growth, and treatment effects^5,8,9^.

The image quality and diagnostic value of computed tomography, particularly CBCT, depend on multiple factors including the tomographic scanner, the imaging parameters (e.g. examination time, tube voltage (kV), tube current (mA), voxel size), the field of view (FOV) and the characteristics of the scanned object itself (e.g. complexity, consistency)^8,10,11^. CBCT is the most widely used 3D radiographic imaging technique in dentistry as it offers lower radiation exposure, lower cost and lower acquisition time than a conventional CT^12^. However, CBCT images often exhibit higher noise, increased scatter, and greater geometric distortions compared to CT scans, potentially compromising image quality^13^. Another limitation of CBCT is the inconsistency of grey scale values, which can be influenced by factors such as the region of interest (ROI) location and FOV size^12,14^. Although a moderate correlation between CBCT grey values and Hounsfield units has been reported, a direct conversion between the two is questionable^15,16^. These aspects must be carefully considered when CBCT data are used for diagnosis, outcome assessment and decision-making, especially concerning the skeletal anatomy^17^.

For various applications requiring the direct representation of skeletal anatomy, a three-dimensional surface model needs to be created from the original tomographic dataset through a process known as segmentation. Previous studies have shown that besides acquisition parameters, bone segmentation can affect the quality of the derived surface models, especially in CBCT images, impacting their diagnostic value^8,18^. The accuracy of the derived 3D surface models defines their appropriateness for applications such as 3D printing of individualised appliances or morphological assessments^18–20^. In tomographic imaging, the obtained benefits need to be weighed against the negative impact of ionizing radiation in an individualised manner. Therefore, assessment of accuracy under different image acquisition settings is crucial, both for determining applicability, and for reducing exposure to radiation. Accuracy assessment consists of trueness and precision (ISO 5727-1), and thus, requires that the true skeletal surface model is available for comparison with the tomographically obtained model. In living patients, such studies would require repeated radiation exposure, including high-radiation images, which is unethical. For this reason, a previous study was based on cadaveric human skulls and established that the accuracy of lower radiation CBCT imaging protocols is similar to conventional CT images and at adequate levels for the assessment of facial morphology^17^. The surface models were acquired with the skulls embedded in water, for soft-tissue simulation, and were compared to those obtained from a highly accurate optical surface scanner^21^, also under hydrated conditions, for comparability^22^.

In the present study we used a similar setting to assess the validity of segmented skeletal surface models of the anterior cranial base from CT and CBCT scans, considering the effect of different scanners, radiation dose protocols and segmentation thresholding. The anterior cranial base consists of thin skeletal structures, and thus, different results from the facial bones could be anticipated. Therefore, the aim of this study was to assess the accuracy level of anterior cranial base 3D surface models generated using different tomographic scanners and acquisition protocols. It was assumed that there were no statistically significant differences between the scanners and protocols.

## Materials and methods

For data generation we used the same materials and adapted methods from Ghamri et al., 2023^17^. Therefore, certain parts of this section are similar to those published previously^17^, but they will be briefly repeated here to illustrate the key elements of our workflow.

### Ethical approval and informed consent

The research ethics committee of the Dental School of the National and Kapodistrian University of Athens (Protocol number: 335, Date of approval: 02/05/2017, Renewed on 16.11.2021) granted ethical approval for the project. All methods were performed in accordance with the relevant guidelines and regulations. Due to the unknown identity of the specimens, a waiver was granted from the ethics committee and no informed consent was traced. Relevant local legislation was respected for all handling of human tissues.

### Material

The study sample consisted of ten human dry skulls, originating from the municipal cemetery of Serres, Greece. Required approval was provided from the local authorities (Municipality of Serres, Greece, Protocol Number: 4044/12.07.2018). The samples were collected for a project focusing on the investigation of 3-dimensional superimposition techniques on skeletal structures of the head^17,21,22^. Included skulls were required to be intact, adult sized, and free from signs of significant aging or pathology, as determined by visual inspection and consensus between two evaluators. As reported previously^22^, the specimens originated from individuals of unknown identity who had died between 8 and 12 years prior to sample collection.

Considering resource availability, along with the authors’ research experience and data, sample size was arbitrarily set. A minimum sample-size of eight specimen was considered adequate^3^. However, a sample size of ten was chosen to facilitate the statistical analysis and add robustness to the findings^21^.

### Image acquisition

Ten direct scans obtained with a structured-light 3D surface scanner and 40 CT/CBCT scans (4 per skull), were acquired from the ten skulls, yielding a total of 50 images for analysis. Similar hydrated conditions were used, to ensure soft-tissue simulation during tomographic image acquisition and account for hydration effects on dimensional integrity^22^ during direct surface scans.

A high-accuracy, optical 3D surface scanner (Artec Space Spider, Artec3D, Luxembourg; Software: Artec Studio 12, Version 12.1.6.16) was used to acquire the ten true reference models. The dry skulls were hydrated by embedding them into room-temperature (22–25 °C) tap water with a pH of approximately 7.5 for 15 min. After this period, they were removed from water, dried by patting them gently with tissue paper, and scanned immediately. The complete sample preparation and scanning protocol has been published previously and showed high precision at about 40 µm^17,21,22^.

A total of 40 CT and CBCT scans were acquired from the same ten specimens over a few days, using four different combinations of scanners and acquisition settings. To simulate soft tissues, each specimen was enclosed in a water-filled 3D printed head shell (PETG, MasterFill Premium PETG Pro, 3DHUB, Greece)^23–25^ (Supplementary Fig. 1). Each specimen underwent four full-head tomographic scans using the following acquisition settings^17^:

1. CT scanner (Revolution CT 256, GE Healthcare, USA; 251 Hellenic Airforce Hospital, Athens, Greece). Tube voltage: 120 kV, tube current: 490 mA in the area of interest (automatically configured based on tissue mass and density), exposure time: 1 s, slice thickness: 0.625 mm, voxel size: 0.49 to 0.62 × 0.49 to 0.62 × 0.31 (interslice) mm, FOV: full head (displayed FOV: 25 cm).
2. CBCT scanner I (Newtom VGiMK4, Verona, Italy; Dental School, National and Kapodistrian University of Athens Greece). Tube voltage: 110 kV, tube current: 4–5 mA (automatically configured based on tissue mass and density), exposure time: 4 s, voxel size: 0.3 × 0.3 × 0.3 mm, FOV: ⌀15 × 15 cm.
3. CBCT scanner II—regular dose settings (Planmeca F, Planmeca Promax 3D Mid 2018, Helsinki, Finland; Digital Iatriki Apeikonisi, Athens, Greece). Tube voltage: 100 kV, tube current: 8 mA, exposure time: 12 s, voxel size: 0.2 × 0.2 × 0.2 mm, FOV: ⌀20 × 17 cm.
4. CBCT scanner II—ultra-low dose settings (Planmeca U). Tube voltage: 100 kV, tube current: 8 mA, exposure time: 6s, voxel size: 0.2 × 0.2 × 0.2 mm, FOV: ⌀20 × 17 cm.

Standardized conditions regarding the water embedding process and the soft-tissue simulation were used for all tomographic scans, which were carried out by professional radiologists. Supplementary Fig. 2 shows example slices of axial reconstructions from each volumetric dataset of one skull.

### Surface model generation

#### Optical surface scanner data

Raw data obtained from the direct surface scans of the skulls were semi-automatically post-processed using Artec Studio 16 software (Version 16.0.5.114, Luxembourg, Luxembourg). In this way, complete full-head surface models were created and saved in Standard Tessellation Language (STL) and Wavefront Object (OBJ) file formats. The complete workflow of the 3D model generation showed very high precision (5–10 µm)^21^. These 10 surface models were considered as the gold standard reference in our study. They were imported into Viewbox 4 software (dHAL Software, Kifissia, Greece) to test the trueness of the corresponding surface models reconstructed from tomographic imaging.

#### Tomographic data

Tomographic data, acquired through CT and CBCT imaging, were exported in DICOM format and imported in Viewbox 4 for further processing. One operator (M.J.), following training in bone segmentation—including detailed demonstrations and supervised practice on multiple samples—extracted the cranial base surface models using a visually defined single threshold. Optimal thresholds were selected through gradual adjustment and enhancement of image contrast. Several 2D tomographic slices were considered to manually adjust and define the threshold isoline that best fitted the outer skeletal surface edge of interest. Selected thresholds were recorded in a Microsoft Excel sheet (Microsoft Corporation, Redmond WA, USA) and dense triangular mesh models were subsequently created with a variant of the marching cubes algorithm^26^. These models were exported and saved as STL files. They consisted of approximately 75,000, 130,000, and 300,000 vertices for the CT, Newtom, and Planmeca generated volumes, respectively.

### Measured outcomes and surface model superimposition

#### Intra and inter-operator reproducibility of the visually defined segmentation threshold

To test the intra-operator reproducibility, the same operator repeated the single-threshold segmentation process of all tomographic images at least 1 week after the first extraction. For inter-operator reproducibility, a second experienced operator (M.G.), who was previously calibrated with the first operator, repeated the segmentation process for four randomly selected tomographic volumes from each setting (16 in total).

#### Intra-operator reproducibility of the visually segmented surface models

**Figure 1:**
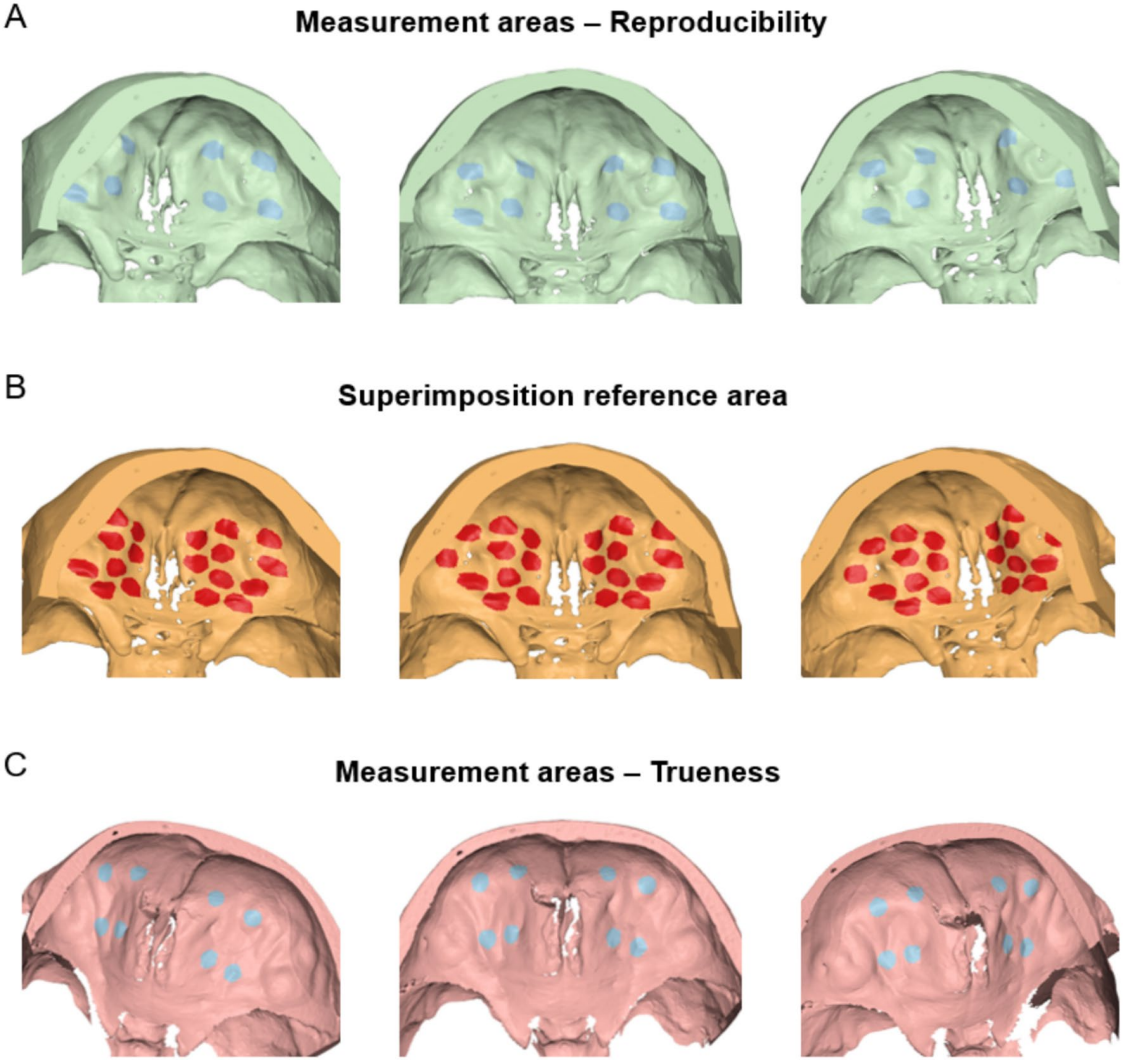
Measurement and superimposition references areas defined on the anterior cranial base. (**A**) Eight circular areas (light blue) selected to measure the reproducibility of the visually segmented surface models. (**B**) Several circular areas (red) evenly distributed across the anterior cranial base selected to superimpose corresponding surface models. (**C**) Eight circular areas (light blue) selected on the true reference surface models to measure the trueness of the tomographically derived surface models. Each circular area consists of 1000 triangles.

Intra-operator reproducibility of the single-threshold segmentation process was assessed at three levels^8^. For the first assessment, the segmented surface models retained their spatial position in the original tomographic volume. The mean absolute distance (MAD) and the standard deviation of the absolute distances (SDAD) of the repeatedly extracted surface models was calculated, based on the distances of each vertex point of one mesh model to the closest point on the other mesh, at eight predefined measurement areas consisting of 1000 triangles each. Measurement areas were selected bilaterally on the anterior cranial base (Fig. 1A). Colour coded distance maps between repeatedly segmented surface models were generated, representing the minimum, average and maximum differences detected in the sample. Following this, repeatedly extracted surface model pairs were superimposed, using a variant of the iterative closest point (ICP) algorithm^27^ with the following parameters: 100% estimated overlap of meshes, matching point to plane, exact nearest neighbour search, 100% point sampling, 50 iterations. The superimposition reference areas were selected bilaterally and distributed evenly across the anterior cranial base, as shown in Fig. 1B. Differences between segmented models in the original position and the position after superimposition were described using the rotational and translational movements required for the best fit matching. Finally, morphological differences, independent of the original position of the models in space, were tested through the MAD and SDAD values between the superimposed models at the predefined measurement areas. Perfect reproducibility was defined as zero MAD prior to superimposition and zero movements/rotations during superimposition^17^. As reproducibility was assessed through repeated segmentations of the same tomographic data, any potential image distortions inherent to the CBCT acquisition remained constant and therefore did not influence the outcome.

#### Trueness of tomographically derived surface models

The tomographically derived surface models were superimposed to their corresponding high-accuracy models, which were directly obtained through the optical surface scanner^21,22^, using the same settings and reference areas described for the intra-operator reproducibility of the segmentation process. As metric to assess trueness, the MAD and SDAD of the superimposed models at eight pre-defined measurement areas, consisting of 1000 triangles each, was calculated. The measurement areas were placed bilaterally on the anterior cranial base of the true reference models (Fig. 1C) ensuring that the corresponding structures were present in the segmented models. Any image distortions inherent to the CBCT acquisition are accounted for in the trueness evaluation, as the segmented models were directly compared to high-accuracy, distortion-free reference scans. Perfect trueness was defined as zero difference between superimposed models. Cases with minimum, average and maximum differences between superimposed models were displayed using colour coded distance maps.

### Statistical analysis

The statistical analysis followed a similar approach to that previously reported^17^ and was conducted using IBM SPSS statistics for Windows, Version 28.0 (Armonk, NY: IBM Corp.). Normality tests were performed using the Shapiro–Wilk test data and visualization of normality plots. Certain variables deviated significantly from normal distribution. Therefore, non-parametric statistics were applied. Differences between repeated identifications of the visually defined segmentation thresholds were visualized through box plots and compared using the Wilcoxon signed-rank test. Differences between acquisition settings were tested using the Kruskal–Wallis test and the overall difference in the amount of error was tested through Mann–Whitney U test. Kruskal–Wallis test was used to test differences between acquisition settings in intra-operator reproducibility of the visually segmented surface models. If applicable, pairwise comparisons were performed through Mann–Whitney U test (significance values adjusted by the Bonferroni correction). All measurement areas selected for each outcome were considered as one variable. Trueness outcomes were tested similarly.

## Results

### Intra and inter-operator reproducibility of the visually defined segmentation threshold

**Figure 2:**
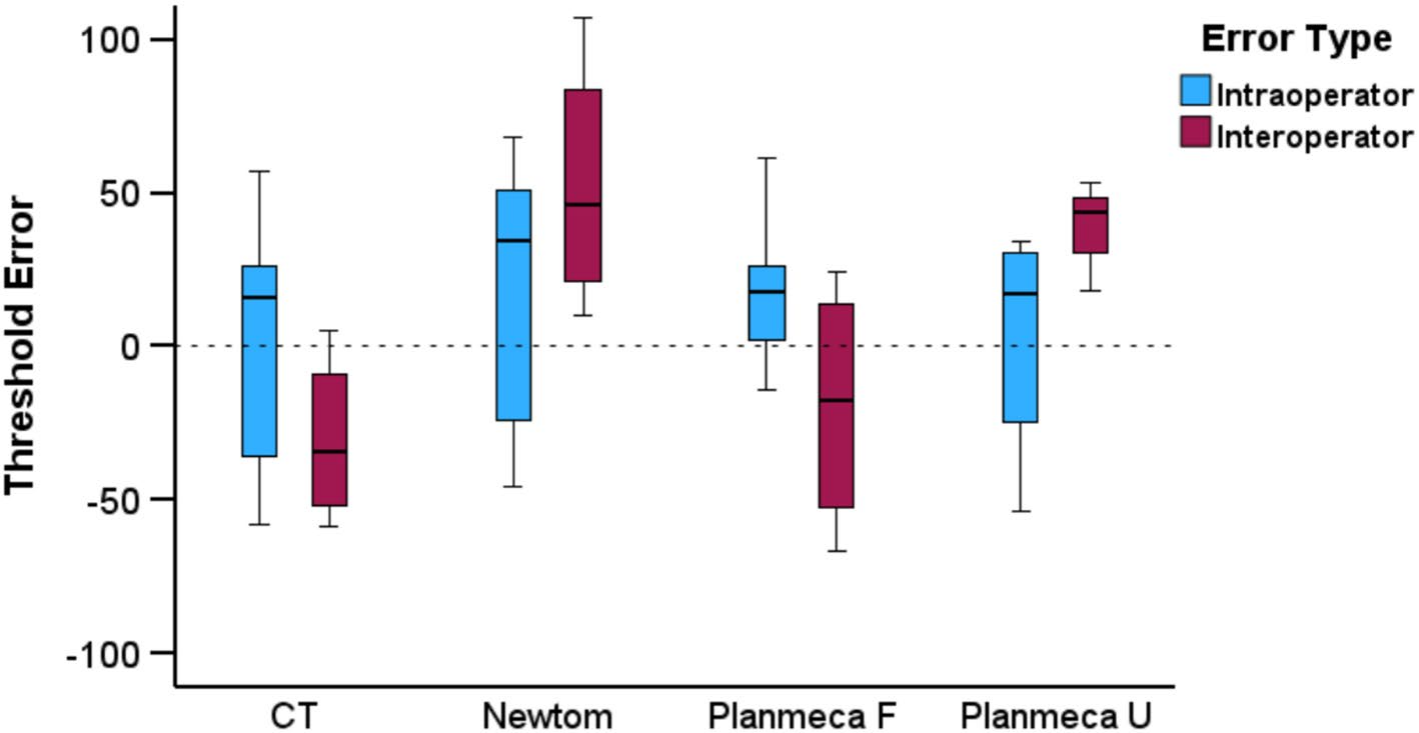
Box plots depicting the intra- and inter-operator error in the determination of visually defined segmentation thresholds (X-axis: tomographic scanners; Y-axis: segmentation threshold values). A difference of 10 in threshold values represents 0.25% of the full range of voxel values for the CT images, 0.22% for the Newtom images, and 0.29% for the Planmeca images.

The median difference between repeated, visually defined segmentation threshold values by the same operator was 21.50 (IQR: 47.75, Wilcoxon signed rank test: *p* = 0.066) and did not differ between acquisition settings (Kruskal–Wallis test: *p* = 0.606). Similar inter-operator outcomes were observed (median: 14.00, IQR: 78.25, Wilcoxon signed rank test: *p* = 0.423). Considering all differences between repeatedly defined thresholds as one variable, similar errors were evident within and between operators (Mann–Whitney U test: *p* = 0.892; Fig. 2).

### Intra-operator reproducibility of the tomographically derived surface models

**Figure 3:**
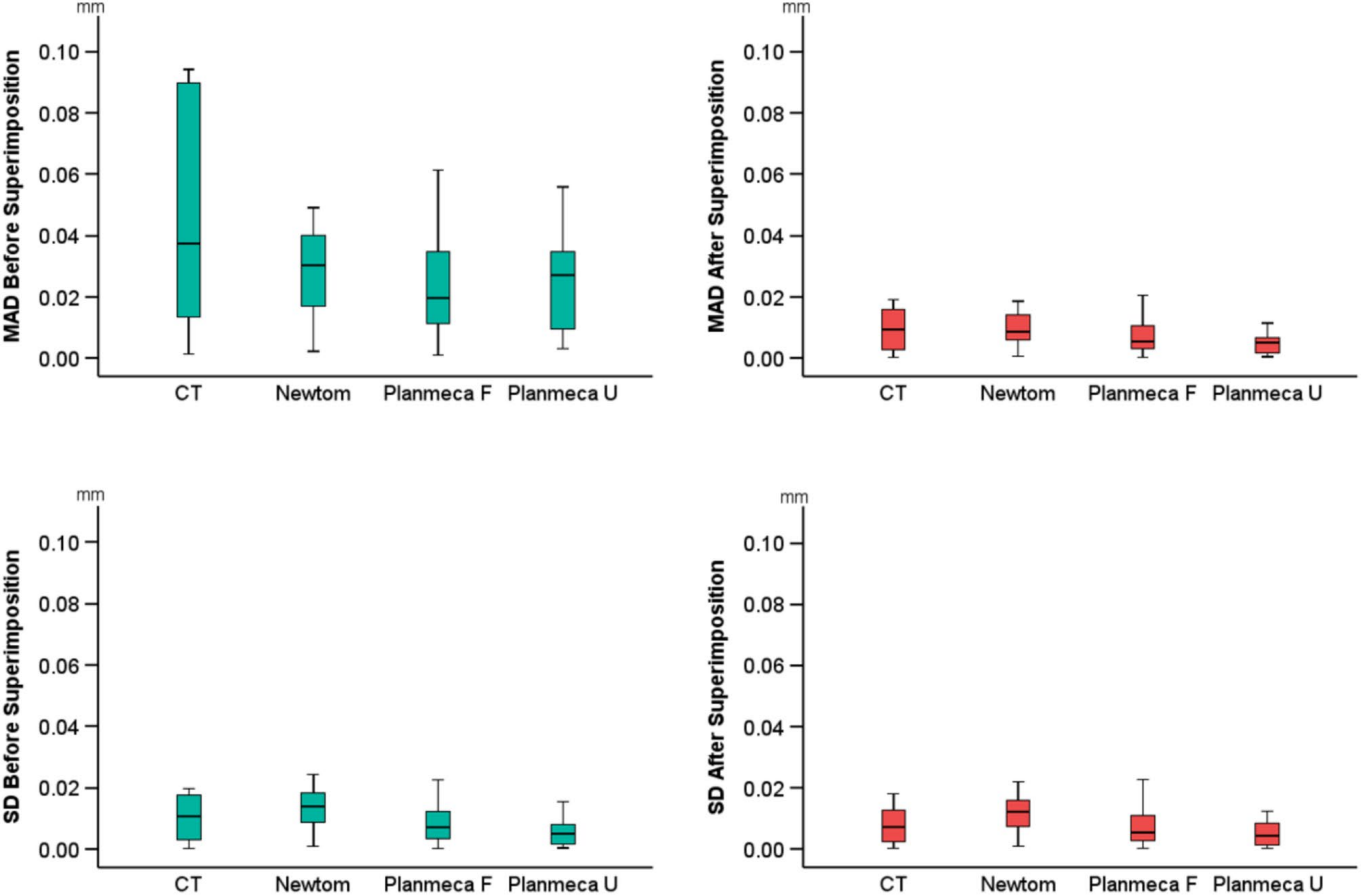
Box plots illustrating the intra-operator differences in repeatedly segmented facial surface models derived from tomographic volumes. The upper graphs display the mean absolute distances (MAD) between the corresponding surface models, while the lower graphs show the standard deviations (SD) of the absolute distances.

A high intra-operator reproducibility of the visually segmented surface models was detected, with a maximum MAD of 0.094 mm and 0.020 mm and a maximum SDAD of 0.024 mm and 0.023 mm, before and after superimposition of the repeatedly segmented models, respectively. The maximum MAD and SDAD before superimposition were detected in a CT scan and a Newtom scan, respectively, whereas the maximum MAD and SDAD after superimposition were both detected in a Planmeca F scan. The reproducibility (MAD) was similar across the different acquisition settings (Kruskall-Wallis test, before superimposition: *p* = 0.626, after superimposition: *p* = 0.423). When the position of the extracted models in space was considered (MAD before superimposition), the CT-derived models showed the highest differences between repeated segmentations (median: 0.037, IQR: 0.079 mm), followed by Newtom (median: 0.030, IQR: 0.024 mm), Planmeca U (median: 0.027, IQR: 0.030 mm) and Planmeca F (median: 0.020, IQR: 0.029 mm). No significant differences were detected when comparing the SDADs among all acquisition settings (Kruskall-Wallis test, before superimposition: *p* = 0.159, after superimposition: *p* = 0.168). The highest SDADs before superimposition were apparent in the Newtom-derived models (median: 0.014, IQR: 0.012 mm), followed by CT (median: 0.011, IQR: 0.014 mm), Planmeca F (median: 0.007, IQR: 0.011 mm) and Planmeca U (median: 0.005, IQR: 0.007 mm) (Fig. 3).

**Figure 4:**
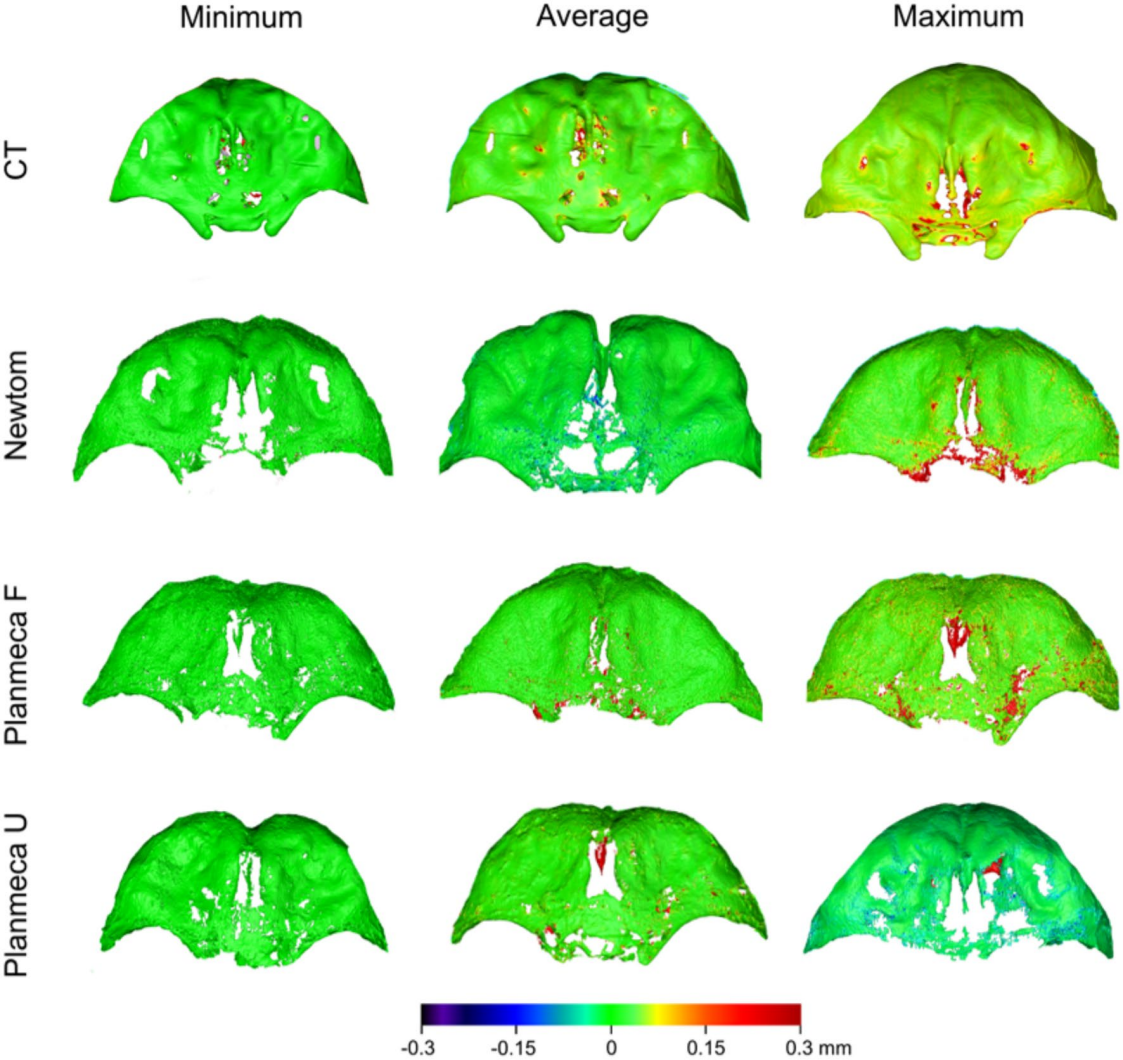
Colour-coded distance maps comparing surface models repeatedly segmented by the same operator, representing the minimum, average, and maximum detected differences. The compared models retained their original positions within the tomographic volume (before superimposition).

Colour-coded distance maps between the surface models at their original position showed equally distributed differences for all acquisition settings, limited within a range of 0.1 mm (Fig. 4). After best-fit approximating the models, the differences were reduced at a level below 0.04 mm (Supplementary Fig. 3).

**Figure 5:**
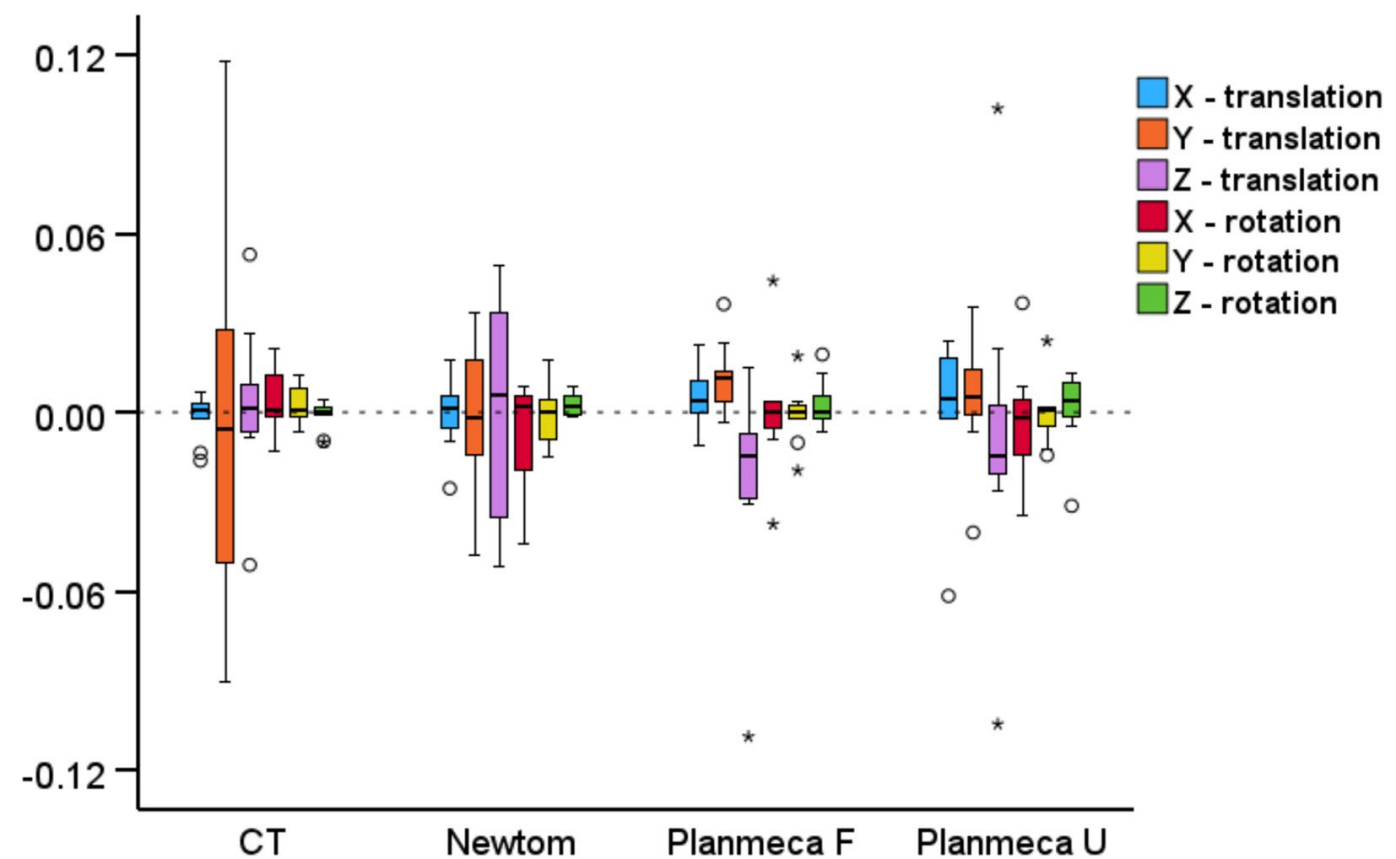
Box plots showing the rotational (°) or translational (mm) movements required to best-fit approximate the repeatedly segmented surface models by the same operator, for each acquisition setting. Outliers are shown as black circles (further from the median more than 1.5 times the IQR) or asterisks (further from the median more than 3 times the IQR). X-translation: lateral movement, Y-translation: vertical movement, Z-translation: anteroposterior movement, X-rotation: around the lateral axis, Y- rotation: around the vertical axis, Z- rotation: around the anteroposterior axis.

Regarding the rotational and translational movements required for the best-fit approximation of the surface models segmented by the same operator, no difference between acquisition settings was found (X-translation: *p* = 0.365, Y-translation: *p* = 0.518, Z-translation: *p* = 0.266, X-rotation: *p* = 0.779, Y-rotation: *p* = 0.890, Z-rotation: *p* = 0.653). The magnitude of translations or rotations was consistently less than 0.12 mm or degrees, respectively, and could therefore be considered as very limited (Fig. 5).

### Trueness of the tomographically derived surface models

**Figure 6:**
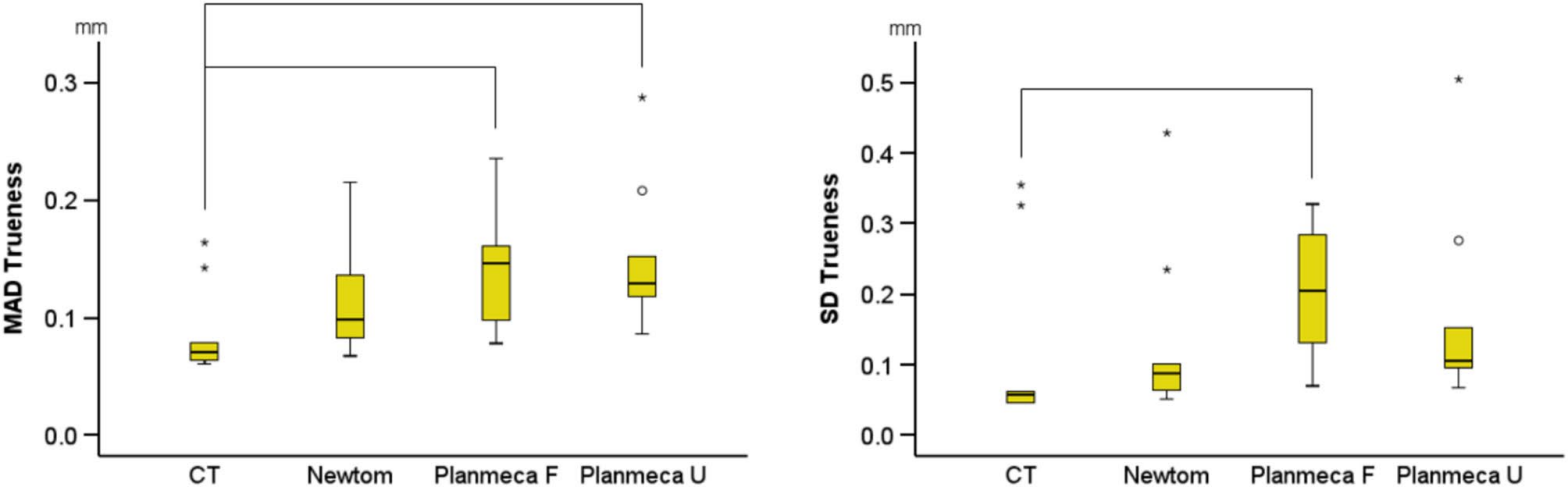
Box plots showing the trueness of the segmented surface models indicated by the distances of the tomographically derived models superimposed with the direct optical scans. The mean absolute distances (MAD) and the standard deviations of the absolute distances (SD) are shown. The lines connect variables that show significant differences (*p* < 0.05) detected through Kruskal–Wallis, followed by Mann–Whitney U test (Bonferroni adjusted). Outliers are shown as black circles (further from the median more than 1.5 times the IQR) or asterisks (further from the median more than 3 times the IQR).

The overall trueness (MAD) was 0.115 mm (IQR: 0.072), with an SDAD of 0.097 (IQR: 0.158), with significant differences between acquisition settings (Kruskal–Wallis Test: *p* = 0.007; CT, median: 0.070, IQR: 0.031; Newtom, median: 0.099, IQR: 0.077; Planmeca F, median: 0.146, IQR: 0.077; Planmeca U, median: 0.129, IQR: 0.051). There were also significant differences between acquisition settings on the SDADs (Kruskal–Wallis Test, *p* = 0.011; CT median: 0.058, IQR: 0.081; Newtom, median: 0.088, IQR: 0.073; Planmeca F, median: 0.206, IQR: 0.172, Planmeca U, median: 0.106, IQR: 0.088) (Fig. 6).

**Figure 7:**
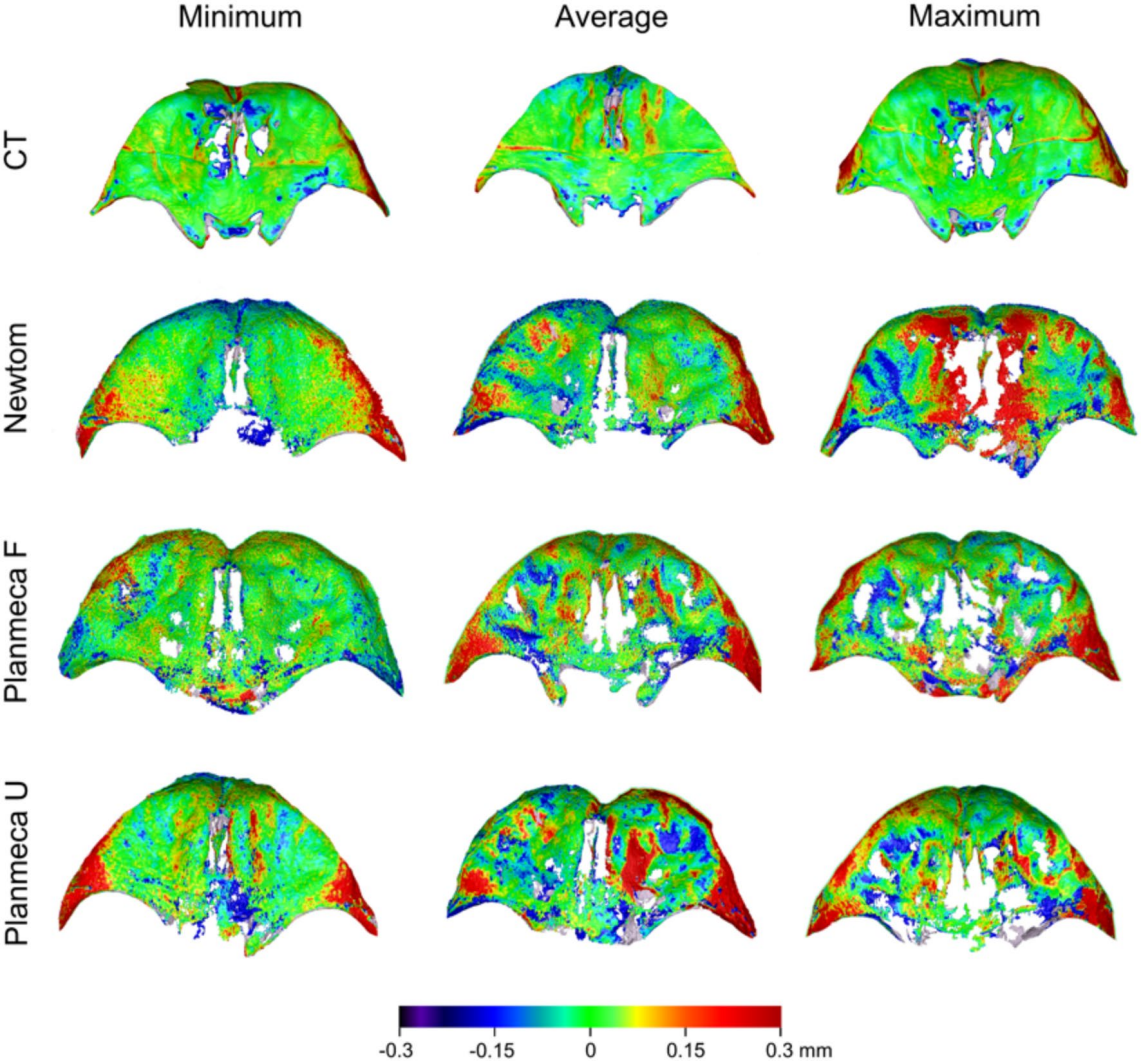
Colour-coded distance maps comparing best-fit approximated segmented surface models with directly obtained models from an optical surface scanner, representing the minimum, average, and maximum deviations in trueness.

Consistent outcomes were revealed by the visualisation of the colour coded distance maps between the best-fit approximated segmented facial models and the directly scanned skulls. The CT models showed higher trueness values, limited within 0.15 mm, whereas the CBCT-derived models showed lower trueness, limited within 0.2 mm, with inaccuracies occupying larger surface areas (Fig. 7).

## Discussion

Accurate 3D imaging is vital for modern diagnostic and treatment planning in medicine and dentistry. This study evaluates the accuracy of CT- and CBCT-derived surface models under realistic clinical conditions, addressing key gaps in prior research. In this study the accuracy of CT- and CBCT-derived ACB surface models was tested by comparing them to respective gold standard reference models. The latter were obtained through direct scans using a high accuracy optical surface scanner. CT and CBCT scanners were used at regular radiation dose settings, as well as at a reduced radiation dose setting, offered by one CBCT scanner. The trueness of segmented surface models derived from various radiographic acquisition protocols has been investigated in previous studies; however, these studies had certain limitations, such as artificially prepared models (rather than real human skulls)^28^, no soft-tissue simulation^29,30^ and imprecise test outcomes, such as inter-landmark distances^10,31^. These limitations and their impact have been highlighted in previous reports^4,18,22,32–34^. The present study approached and improved upon these limitations by simulating real-life conditions regarding soft-tissue effects^22^ and tomographic settings, incorporated accurate true reference models^21^ and accounted for the actual morphology of the entire anterior cranial base for outcome assessment.

We found that accurate 3D surface models of the anterior cranial base could be generated using various tomographic scanners and techniques (CT/CBCT), even with a low-dose protocol. An average trueness of 0.115 mm was observed across all acquisition methods, with no clinically relevant differences between scanners or settings. Colour-coded distance maps demonstrated robust models in all cases with deviations extending up to 0.2 mm in certain areas. This finding is highly significant, as the anterior cranial base is a key area of interest in multiple clinical fields (e.g., orthodontics and cranio-maxillofacial surgery), as well as in various disciplines of biology and anthropology^1,6,35^. Accurate 3D surface models of this region are essential for studying craniofacial morphology, enabling proper diagnosis, treatment outcome assessment, and growth evaluation^18,19^. They are also crucial for planning surgical procedures^10^ and manufacturing cranio-maxillofacial prostheses^20^. However, these findings do not apply to assessments of micro-morphology, bone density, or other fine-scale structural characteristics.

Recently, using a similar methodology, our team found consistent results on the accuracy of 3D skeletal surface models of the face, demonstrating their sufficiency for overall morphology assessment^17^. These findings support accurate craniofacial morphology assessment via ACB surface model superimposition. The anterior cranial base serves as the primary craniofacial superimposition reference^3,36,37^, due to its anatomical stability during growth^38^ and its central position within the craniofacial complex^35^. On the other hand, facial appearance has a significant impact on various life outcomes, including employability and both social and personal relationships^39–41^. If the 3D morphology of these key areas is accurately captured in segmented surface models from tomographic volumes, surface-based superimposition enables precise and efficient 3D analysis of craniofacial changes over time. However, direct assessment of ACB surface superimposition accuracy for evaluating facial outcomes is currently unavailable. The reliability of surface models generated with low-radiation protocols for craniofacial morphology assessment is promising for the broader adoption of tomographic imaging. Combined with other radiation reduction strategies—such as limiting the field of view, using protective shields, and advancing software and hardware^3,42,43^—this may improve the cost–benefit ratio for patients.

An alternative to surface-segmentation methods is voxel superimposition, based directly on the raw voxel data^4,5,36^. However, software support is limited and demarcation of the area of interest is constrained to rectangular areas that do not conform to bone anatomy. Additionally, 3D surface models are usually required in any case for a realistic 3D assessment, so surface-based superimposition, which can provide accurate outcomes using appropriate registration settings^44–46^, seems a preferred choice.

Segmentation errors can result in surface models that deviate from the actual morphology. Previous studies have shown that the threshold used for bone segmentation from tomographic volumes can influence the generated surface model^8^ and that even minor artifacts can impact the accuracy of surface-based superimposition^47^. We employed a visually defined single-threshold approach for bone segmentation. The inherent limitations of this approach, particularly for CBCT images, have been discussed in previous studies, as grayscale values for similar structures may vary depending on their position within the tomographic volume^12,14,48^. Alternative methods overcoming these problems have been suggested^49–51^. Nevertheless, the single-threshold approach remains the standard method for CBCT segmentation due to its simplicity and availability in commonly used software. It has shown comparable outcomes to the time-consuming manual segmentation approach^8^ and has demonstrated reliability when compared to gold-standard reference models in both a previous study^17^ and the present one. In the present study, the segmentation error was minimal and consistent across various acquisition protocols. Findings on differences between repeatedly segmented models support this, suggesting that segmentation is a negligible source of error in the overall outcome^17,18,52^. However, all the aforementioned studies focused on bone segmentation for outcome assessment rather than its impact on surface-based superimposition outcomes.

Room-temperature tap water was used as a single-material embedment for soft-tissue simulation. Soft tissues exhibit varying densities, making this approach not fully representative of actual conditions. Nevertheless, single materials such as wax sheets, water, or gel-like materials have been shown to adequately simulate soft-tissues in similar experimental settings^23,24,53^. Water allows multiple tomographic scans without compromising specimen integrity. Previous studies indicate that hydrating dry bones alters their anatomical form^22,33^. Therefore, all dry skulls were hydrated before true-reference acquisition with the optical surface scanner to ensure model comparability. Another strength of this study is the use of CT and CBCT imaging protocols established by specialist radiologists, following standard practices for craniofacial morphology assessment. Additionally, thorough testing of the imaging, superimposition, and assessment methods enhances the reliability of the findings^3,5,8,18,21,22,36,54^.

Despite its several strengths—including realistic simulation of clinical conditions, testing of different scanners and acquisition protocols, use of a validated true reference model for comparisons, and precise implementation and reporting of established 3D imaging analysis methods—this study also has certain limitations. One such limitation is that motion-related artifacts, which commonly occur in real-world clinical imaging involving live patients, were not present due to the use of human skulls^55^. Another limitation is that the algorithm used measures the distance between selected vertices on one surface to the nearest point on the other, which may not be anatomically correspondent. As a result, the calculated surface mesh distances may slightly underestimate the true anatomical difference. Finally, age information for the sample was unavailable. While most skulls appeared to belong to adults, a specific age range could not be determined. This did not affect internal comparisons, as the same skulls were used across all methods, nor did it compromise the robustness of the outcomes. However, it limits the study’s generalizability to very young individuals.

## Conclusions

The results of this study confirm that reduced-radiation CBCT imaging protocols can produce 3D surface models of the anterior cranial base with sufficient accuracy for craniofacial morphology assessment and related applications, comparable to conventional CT images. Furthermore, the study demonstrated that lower-radiation CBCT protocols perform comparably to standard CBCT protocols for similar purposes. Statistically significant differences were observed between the tomographic scanners and acquisition protocols; however, these appear to be clinically irrelevant. These findings support a more favourable cost–benefit ratio regarding radiation exposure, thereby enhancing patient safety.

## Supplementary Information

Supplementary Information.

## Data Availability

All data are available in the main text or the extended data. The datasets generated and/or analysed during the current study are available on request from the corresponding author. Due to the sensitive nature of the used specimens, the raw data remain confidential and cannot be shared.
