# Supplementary material for: Accuracy of anterior cranial base surfaces acquired from computed tomography imaging
Source: Sci Rep. 2025 Jul 7;15:24321. doi: 10.1038/s41598-025-09104-w (PMC12234761; doi:10.1038/s41598-025-09104-w)
Supplement: Supplementary file 1 — Supplementary Information. [file 41598_2025_9104_MOESM1_ESM.pdf]

Supplementary material

Accuracy of anterior cranial base surfaces acquired from computed tomography imaging.

Maurus Kurt Jaeggi, Mohammed Ghamri, Konstantinos Dritsas, Simos Psomiadis, Carlalberta Verna, Demetrios Halazonetis, Nikolaos Gkantidis

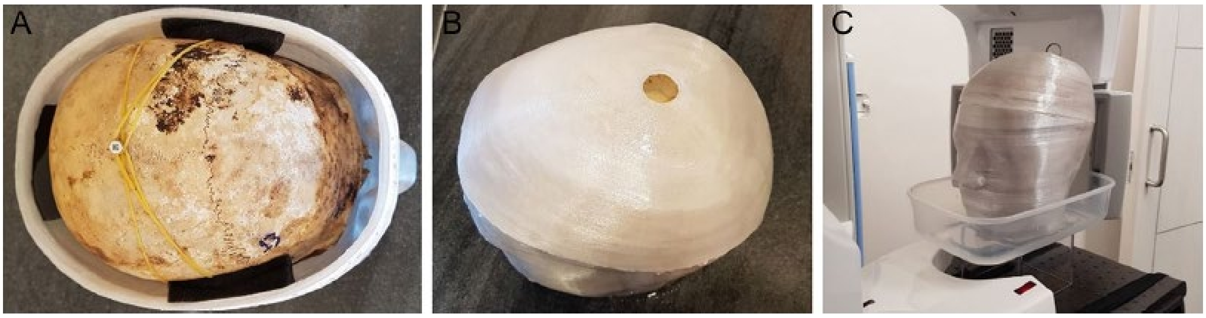

Supplementary Figure 1. (A). Final configuration of an entire skull subjected to tomographic imaging. (B) The 3D printed soft-tissue head shell was filled with water, through the hole present at its upper part. (C). Tomographic image acquisition with soft-tissue simulation. *Reprint from Ghamri et al. Scientific Reports (2023) 13:21002, https://doi.org/10.1038/s41598-023-48320-0.*

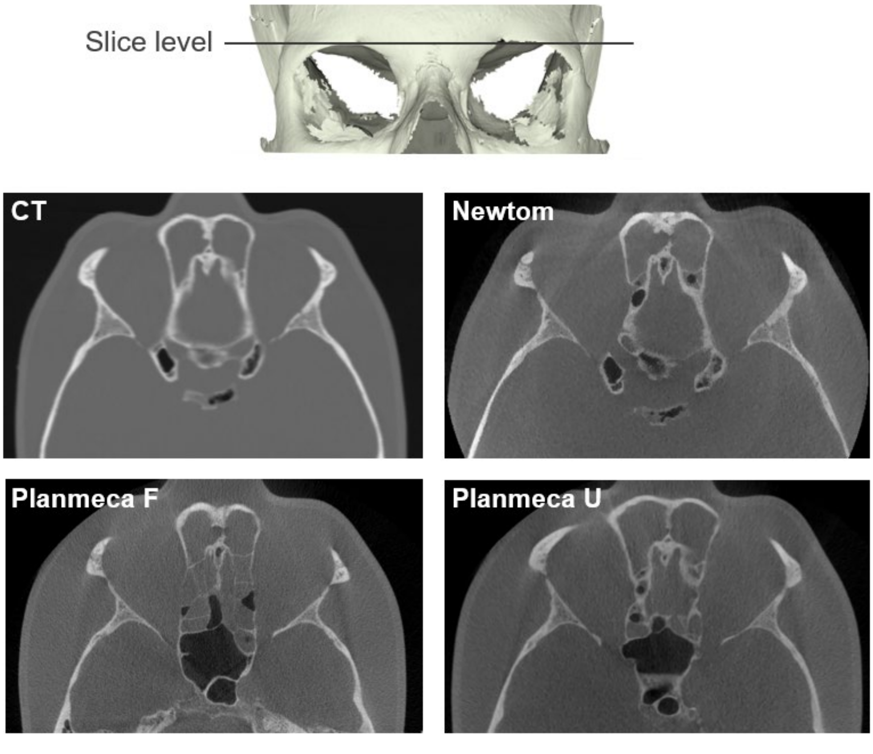

Supplementary Figure 2. Sample axial reconstructions of tomographic volumes obtained from the same skull, through each acquisition setting, at the level of the zygomatic arch.

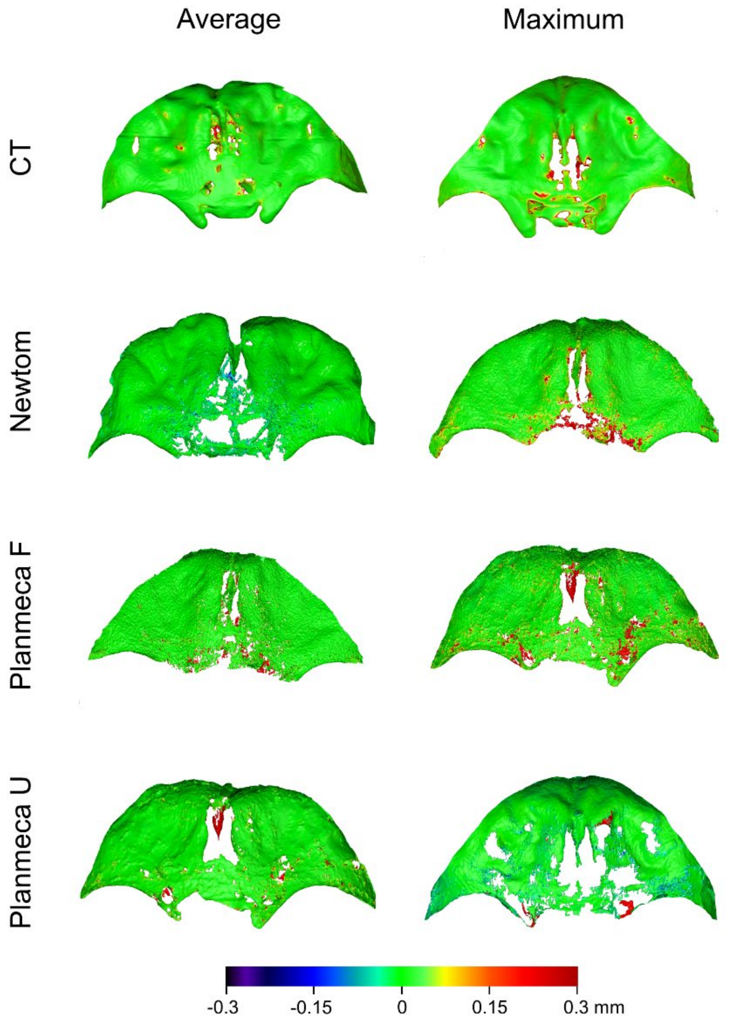

Supplementary Figure 3. Color-coded distance maps comparing repeatedly segmented surface models by the same operator, following their best-fit approximation. The average and maximum detected differences are depicted.
